# Quantitative radiomics analysis of imaging features in adults and children Mycoplasma pneumonia

**DOI:** 10.3389/fmed.2024.1409477

**Published:** 2024-05-20

**Authors:** Huan Meng, Tian-Da Wang, Li-Yong Zhuo, Jia-Wei Hao, Lian-yu Sui, Wei Yang, Li-Li Zang, Jing-Jing Cui, Jia-Ning Wang, Xiao-Ping Yin

**Affiliations:** ^1^Clinical Medicine School of Hebei University, Baoding, China; ^2^Department of Radiology, Affiliated Hospital of Hebei University, Baoding, China; ^3^Hebei Key Laboratory of Precise Imaging of Inflammation Related Tumors, Baoding, China; ^4^Department of Radiology, Baoding First Central Hospital, Baoding, China; ^5^Department of Radiology, Baoding Children's Hospital, Baoding, China; ^6^Department of Research and Development, United Imaging Intelligence (Beijing) Co., Beijing, China

**Keywords:** mycoplasma pneumonia, radiomics, adults, children, computed tomography (CT)

## Abstract

**Purpose:**

This study aims to explore the value of clinical features, CT imaging signs, and radiomics features in differentiating between adults and children with Mycoplasma pneumonia and seeking quantitative radiomic representations of CT imaging signs.

**Materials and methods:**

In a retrospective analysis of 981 cases of mycoplasmal pneumonia patients from November 2021 to December 2023, 590 internal data (adults:450, children: 140) randomly divided into a training set and a validation set with an 8:2 ratio and 391 external test data (adults:121; children:270) were included. Using univariate analysis, CT imaging signs and clinical features with significant differences (*p* < 0.05) were selected. After segmenting the lesion area on the CT image as the region of interest, 1,904 radiomic features were extracted. Then, Pearson correlation analysis (PCC) and the least absolute shrinkage and selection operator (LASSO) were used to select the radiomic features. Based on the selected features, multivariable logistic regression analysis was used to establish the clinical model, CT image model, radiomic model, and combined model. The predictive performance of each model was evaluated using ROC curves, AUC, sensitivity, specificity, accuracy, and precision. The AUC between each model was compared using the Delong test. Importantly, the radiomics features and quantitative and qualitative CT image features were analyzed using Pearson correlation analysis and analysis of variance, respectively.

**Results:**

For the individual model, the radiomics model, which was built using 45 selected features, achieved the highest AUCs in the training set, validation set, and external test set, which were 0.995 (0.992, 0.998), 0.952 (0.921, 0.978), and 0.969 (0.953, 0.982), respectively. In all models, the combined model achieved the highest AUCs, which were 0.996 (0.993, 0.998), 0.972 (0.942, 0.995), and 0.986 (0.976, 0.993) in the training set, validation set, and test set, respectively. In addition, we selected 11 radiomics features and CT image features with a correlation coefficient r greater than 0.35.

**Conclusion:**

The combined model has good diagnostic performance for differentiating between adults and children with mycoplasmal pneumonia, and different CT imaging signs are quantitatively represented by radiomics.

## 1 Introduction

Mycoplasma pneumonia (MP) accounts for 10%–30% of community-acquired pneumonia (CAP) and often occurs in autumn, especially in children and adolescents (1). In recent years, the adult incidence rate has also increased. This disease spreads approximately every 3–7 years (2). During the epidemic period, this microbe can cause up to 20%–40% of CAP cases in the general population and even up to 70% in closed populations (3). The diagnosis of mycoplasmal pneumonia depends on the detection of specific antibodies. Due to its often negative early diagnosis, computed tomography (CT) imaging plays an important guiding role in the early diagnosis and treatment of mycoplasmal pneumonia. Previous studies have shown that children tend to present with large patchy consolidation on CT imaging compared to adults (4), but the discovery of this difference often depends on the reading habits and clinical experience of the reader. Moreover, as the disease progresses, the imaging manifestations at different stages of the same disease are not always the same, and there are often overlapping manifestations. In recent years, the term radiomics has received increasing attention (5). Radiomics has been successfully applied in the identification, staging, and evaluation of lung cancer (6, 7). However, radiomics methods are relatively less applied in the prediction and diagnosis of non-tumor diseases of the lung. Yanling et al. (8) applied radiomics nomograms to identify pneumonia and acute paraquat lung injury. Xie et al. (9) applied CT radiomics to conduct a comparative analysis of ground-glass density shadows in COVID-19 and non-COVID-19 and proposed that the CT radiomics model can help to differentiate between COVID-19 and non-COVID-19 ground-glass density shadows. At the same time, Honglin Li et al. (10) confirmed that radiomics-clinical nomograms have good discriminative effects on mycoplasmal pneumonia and bacterial pneumonia, which is helpful for clinical decision-making. In addition, radiomics also plays an important role in grading severity (11) and prognostic evaluation (12) of pneumonia.

The above results provide confidence and reference for our research. Considering that there is no research on differentiating the radiomic features of adult and child mycoplasmal pneumonia in domestic and foreign studies, this article will analyze and compare the clinical features, CT imaging signs, and radiomic features of adult and child patients and conduct external validation. It will provide a quantitative representation of different CT imaging signs using radiomics, thus providing evidence for early clinical diagnosis and precise treatment.

## 2 Materials and methods

### 2.1 Study population

**Figure 1:**
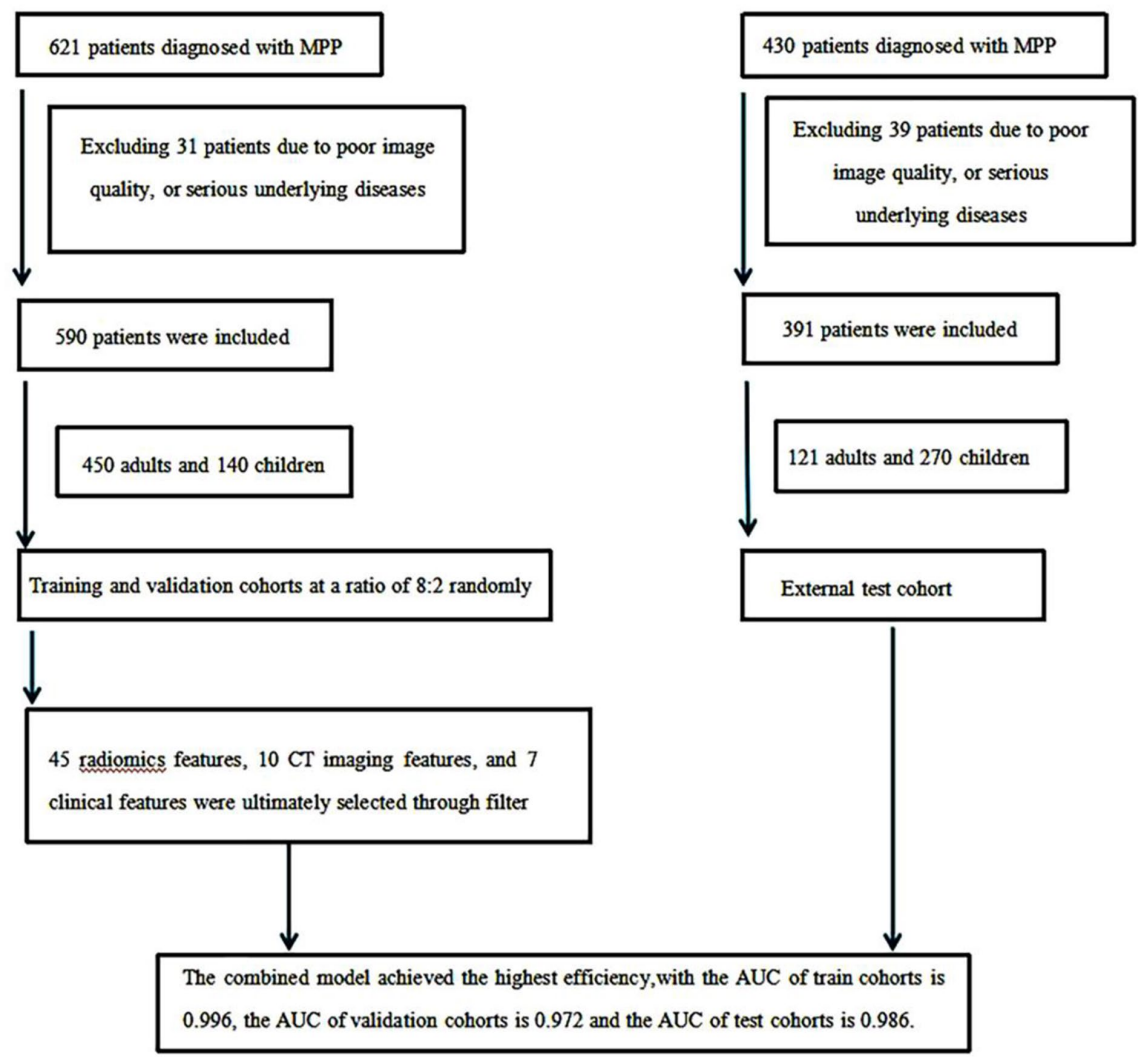
Research flowchart.

In a retrospective analysis of clinical and imaging data of patients diagnosed with MP in two hospitals from November 2021 to December 2023, 590 patients (450 adults and 140 children) with internal data, which were divided into a training set and a validation set according to an 8:2 ratio, and 391 patients (121 adults and 270 children) with external data, which were used as an external test set, were included. Based on age, patients were divided into adult group (>14 years old) and child group (≤14 years old). The inclusion criteria were as follows: (1) patients with mycoplasmal pneumonia confirmed by throat swab or fiberoptic bronchoscopy with alveolar lavage nucleic acid testing; and (2) patients with clear lesions detected by chest CT. Exclusion criteria were as follows: (1) poor image quality; and (2) previous bronchial asthma, chronic obstructive pulmonary disease, recurrent respiratory tract infections, severe pneumonia without a history of cure, congenital or secondary immune suppression or immune deficiency, and connective tissue disease (Figure 1). This study was approved by the ethics committee of the Affiliated Hospital of Hebei University, and because this is a retrospective study, written informed consent is waived. This study was conducted in accordance with the principles of the Helsinki Declaration.

### 2.2 CT image acquisition

Philips Brilliance 256-row, GE Discovery HD750 CT, and United Imaging uCT550 spiral CT scanner were used. The patient was placed in a supine position with both hands raised above his head. The scanning range was from the thoracic inlet to the level of the diaphragm, and deep breath-holding scanning was performed after deep inspiration. Scanning parameters: tube voltage 120 kV, tube current automatic milliamp technology, pitch 0.900; 0.984; 1.175, Rotation time 0.5; 0.6; 0.6 s, matrix 512 × 512, layer thickness 5 mm, interlayer spacing 5 mm, and field of view 40 cm × 40 cm. Axial reconstruction of lung window (window width 1500HU, window level −600 HU) and mediastinal window (window width 350HU, window level 40HU).

### 2.3 CT image analysis

The CT images were independently reviewed by two physicians mainly engaged in chest imaging diagnosis. In case of disagreement, the two physicians reached a consensus through consultation. The CT characteristics of each patient were recorded, including consolidation pattern, consolidation with ground-glass opacity (GGO), bronchial wall thickening, air bronchogram, atelectasis, interlobular septal thickening, number of involved lung lobes, mediastinal enlargement of lymph nodes, pleural effusion, and other imaging features, as well as quantitative characteristics such as mean lesion density, lesion volume, and CTLP.

### 2.4 Radiomics feature extraction, feature selection, and machine learning models building

**Figure 2:**
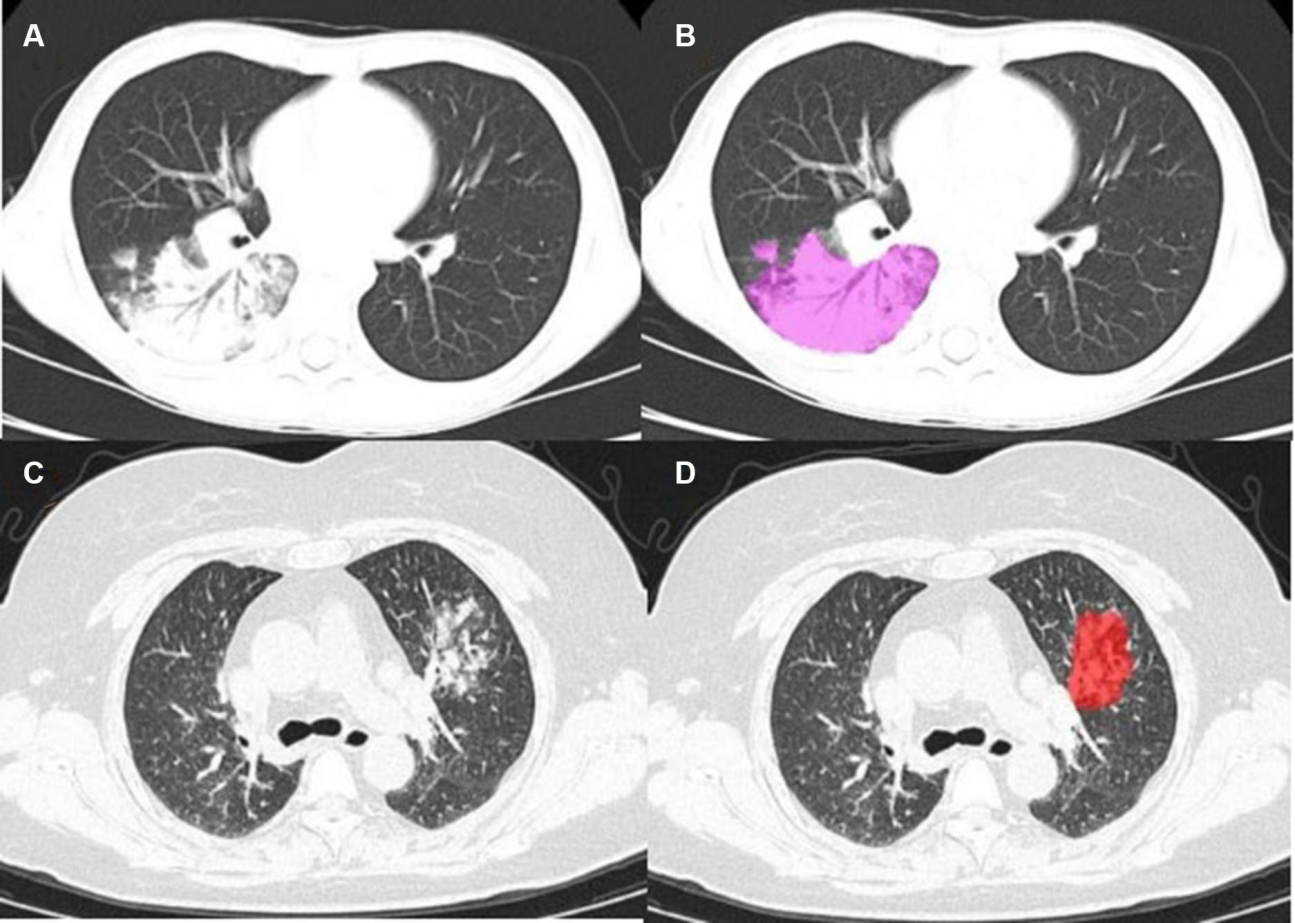
**(A)** Mycoplasmal pneumonia in a child (male, 9 years old), mainly manifested as large patchy consolidation, with air bronchogram sign visible; **(C)** Mycoplasmal pneumonia in an adult (female, 57 years old), characterized by focal and small patchy consolidation; **(B–D)** Lesion annotation.

Before radiomics feature extraction, the images were normalized by subtracting the window level (WL: 40) and dividing by the window width (WW: 300). The auto-segmentation, radiomics feature extraction, feature selection, and machine learning models building were established on the uAI Research Portal V1.1 (Shanghai United Imaging Intelligence, Co., Ltd.) (13–16). The radiomics features were automatically extracted from ROIs using an open-source Python package, Pyradiomics V3.0 (17). The PCC, LASSO, LR, and other methods used the package of Scikit-learn (18). All analyses were implemented in Python (Python Software Foundation, http://python.org). Two physicians modified the ROI of the automatically segmented lesions layer-by-layer to avoid non-lesion areas such as blood vessels and ribs, confirmed and submitted it, and obtained the volume of interest (VOI) of the lesion (Figure 2). The features were divided into seven groups, and the shape features were extracted from the original image based on the ROI. The texture features and grayscale statistics features were extracted from the original image and 15 filtered images, with a total of 1,904 features extracted, which were:

1. Shape feature: 14;
2. Grayscale statistics feature: 450;
3. Gray Level Cooccurence Matrix, GLCM: 525;
4. Gray Level Run Length Matrix, GLRLM: 350;
5. Gray Level Size Zone Matrix, GLSZM: 400;
6. Neighboring Gray Tone Difference Matrix, NGTDM: 400;
7. Gray Level Dependence Matrix, GLDM: 125.

Using univariate analysis to select CT imaging signs and clinical features with significant differences (*p* < 0.05), we constructed the clinical model. Z-score was used to normalize radiomics features before feature selection and model construction. Pearson correlation coefficient (PCC) and least absolute shrinkage and selection operator (LASSO) were used to screen and reduce the dimensionality of radiomic features, and RadScore was calculated by weighting the features based on the coefficients obtained by LASSO. In addition, multivariable logistic regression analysis is used to construct radiomic models based on the features selected. Finally, the combined model was constructed using Radscore, CT imaging signs, and clinical features selected.

Importantly, the radiomics features and quantitative and qualitative CT imaging signs were analyzed using Pearson correlation analysis and analysis of variance, respectively.

### 2.5 Statistical analysis

All data were analyzed using SPSS 26.0. For quantitative data, independent sample t-tests (when normal distribution) or Mann–Whitney U-tests (when non-normal distribution) were performed. For count data, χ2 tests were performed. Logistic regression analysis was performed on the clinical features, CT imaging signs, and radiomics features that showed statistical differences between the groups. Single-phase models and combined models were established, and the predictive performance of each model was evaluated using AUC, sensitivity, specificity, and accuracy. The Delong test was used to compare the AUCs between the models. Pearson correlation analysis and variance analysis were used to analyze quantitative and qualitative CT imaging signs and radiomics features, respectively, to find the quantitative radiomics of CT imaging signs.

## 3 Results

### 3.1 Clinical features

Statistical analysis was conducted on the clinical data of the training set, validation set, and test set. There were significant differences in the type of fever, LC, CK-MB, LDH, D-dimer, and CRP between adult and child groups with mycoplasmal pneumonia (*p* < 0.05), but there was no significant difference in PLT. The proportion of severe cases in the training set was 30.8% in the adult group and 36.6% in the child group; in the validation set, it was 32.2% in the adult group and 35.7% in the child group; in the test set, it was 21.5% in the adult group and 36.7% in the child group, with statistically significant differences (*p* < 0.05). The details are shown in Table 1.

**Table 1:** General information of adult and child patients with mycoplasmal pneumonia.

	Train *N* = 472			Validation *N* = 118			Test *N* = 391			Overall *N* = 981
Characteristic	0	1	*p*-value^1^	0	1	*p*-value^1^	0	1	*p*-value^1^	*p*-value^2^
	*N* = 360(76%)	*N* = 112(24%)		*N* = 90(76%)	*N* = 28(24%)		*N* = 121(31%)	*N* = 270(69%)		
Age	58 (17)	7 (3)	<0.001	57 (16)	7 (4)	<0.001	46 (19)	7 (3)	<0.001	<0.001
Gender			0.48			0.88			0.043	0.033
Female	200 (55.6%)	58 (51.8%)		40 (44.4%)	12 (42.9%)		48 (39.7%)	137 (50.7%)		
Male	160 (44.4%)	54 (48.2%)		50 (55.6%)	16 (57.1%)		73 (60.3%)	133 (49.3%)		
Type_of_fever			<0.001			<0.001			<0.001	<0.001
None	276 (76.7%)	19 (17.0%)		69 (76.7%)	8 (28.6%)		26 (21.5%)	4 (1.5%)		
Grade 1(37.1–38°C)	24 (6.7%)	6 (5.4%)		6 (6.7%)	0 (0.0%)		24 (19.8%)	8 (3.0%)		
Grade 2(38.1–39°C)	35 (9.7%)	38 (33.9%)		11 (12.2%)	8 (28.6%)		50 (41.3%)	103 (38.1%)		
Grade 3(39.1–41°C)	25 (6.9%)	48 (42.9%)		4 (4.4%)	12 (42.9%)		21 (17.4%)	153 (56.7%)		
Grade 3(>41°C)	0 (0.0%)	1 (0.9%)					0 (0.0%)	2 (0.7%)		
LC	1.56 (1.14,2.03)	2.16 (1.61,2.88)	<0.001	1.51 (1.14,2.06)	2.43 (1.63,4.05)	<0.001	1.50 (1.10,2.01)	2.12 (1.61,2.70)	<0.001	<0.001
PLT	247 (192,312)	298 (236,383)	<0.001	245 (193,329)	300 (255,368)	0.001	256 (195,309)	284 (228,354)	<0.001	0.031
CK-MB	0.70 (0.40,1.10)	1.10 (0.60,2.20)	<0.001	0.60 (0.39,0.91)	1.51 (0.60,2.22)	0.001	12.8 (10.7,17.3)	2.3 (1.9,2.9)	<0.001	<0.001
LDH	191 (161,231)	261 (222,323)	<0.001	188 (155,227)	277 (206,337)	<0.001	173 (127,211)	284 (242,336)	<0.001	<0.001
D-dimer	178 (137,343)	137 (1,235)	<0.001	162 (137,298)	137 (104,234)	0.064	1.31 (0.42,9.77)	0.23 (0.15,0.38)	<0.001	<0.001
CRP	6 (2,30)	6 (1,19)	0.13	6 (2,41)	7 (2,18)	0.43	19 (6,48)	7 (2,16)	<0.001	0.024
Severe			0.25			0.73			0.003	0.98
No	249(69.2%)	71(63.4%)		61(67.8%)	18(64.3%)		95(78.5%)	171(63.3%)		
Yes	111(30.8%)	41(36.6%)		29(32.2%)	10(35.7%)		26(21.5%)	99(36.7%)		
Consolidation_pattern			0.001			0.86			<0.001	<0.001
None	134 (37.2%)	26 (23.2%)		33 (36.7%)	9 (32.1%)		28 (23.1%)	41 (15.2%)		
Patchy	77 (21.4%)	16 (14.3%)		16 (17.8%)	7 (25.0%)		42 (34.7%)	83 (30.7%)		
Segmental	77 (21.4%)	38 (33.9%)		27 (30.0%)	8 (28.6%)		40 (33.1%)	63 (23.3%)		
Wedge-shaped	72 (20.0%)	32 (28.6%)		14 (15.6%)	4 (14.3%)		11 (9.1%)	83 (30.7%)		
Consolidation_mixed_GGO			<0.001			0.026			0.003	<0.001
No	253 (70.3%)	58 (51.8%)		68 (75.6%)	15 (53.6%)		70 (57.9%)	113 (41.9%)		
Yes	107 (29.7%)	54 (48.2%)		22 (24.4%)	13 (46.4%)		51 (42.1%)	157 (58.1%)		
Pleural_effusion			<0.001			0.24			0.005	
None	276 (76.7%)	107 (95.5%)		71 (78.9%)	27 (96.4%)		101 (83.5%)	253 (93.7%)		
Minor	58 (16.1%)	4 (3.6%)		8 (8.9%)	1 (3.6%)		15 (12.4%)	11 (4.1%)		
Moderate	14 (3.9%)	1 (0.9%)		8 (8.9%)	0 (0.0%)		4 (3.3%)	3 (1.1%)		
Massive	12 (3.3%)	0 (0.0%)		3 (3.3%)	0 (0.0%)		1 (0.8%)	3 (1.1%)		
Mediastinal_enlargement_of_lymph_nodes			0.001			0.020			0.002	0.004
No	295 (81.9%)	106 (94.6%)		75 (83.3%)	28 (100.0%)		104 (86.0%)	257 (95.2%)		
Yes	65 (18.1%)	6 (5.4%)		15 (16.7%)	0 (0.0%)		17 (14.0%)	13 (4.8%)		
Air_bronchogram_sign			0.003			0.25			<0.001	<0.001
No	228 (63.3%)	53 (47.3%)		56 (62.2%)	14 (50.0%)		63 (52.1%)	89 (33.0%)		
Yes	132 (36.7%)	59 (52.7%)		34 (37.8%)	14 (50.0%)		58 (47.9%)	181 (67.0%)		
bronchial_wall_thickening			0.010			0.29			0.93	0.28
No	247 (68.6%)	91 (81.3%)		70 (77.8%)	19 (67.9%)		83 (68.6%)	184 (68.1%)		
Yes	113 (31.4%)	21 (18.8%)		20 (22.2%)	9 (32.1%)		38 (31.4%)	86 (31.9%)		
Interlobular_septal_thickening			<0.001			0.029			0.019	<0.001
No	244 (67.8%)	102 (91.1%)		66 (73.3%)	26 (92.9%)		94 (77.7%)	235 (87.0%)		
Yes	116 (32.2%)	10 (8.9%)		24 (26.7%)	2 (7.1%)		27 (22.3%)	35 (13.0%)		
Number_of_lobes_involved						0.40			<0.001	
1	133 (36.9%)	60 (53.6%)		28 (31.1%)	14 (50.0%)		29 (24.0%)	97 (35.9%)		
2	37 (10.3%)	25 (22.3%)		12 (13.3%)	4 (14.3%)		22 (18.2%)	65 (24.1%)		
3	50 (13.9%)	6 (5.4%)		10 (11.1%)	2 (7.1%)		16 (13.2%)	52 (19.3%)		
4	42 (11.7%)	7 (6.3%)		9 (10.0%)	2 (7.1%)		17 (14.0%)	20 (7.4%)		
5	93 (25.8%)	8 (7.1%)		27 (30.0%)	4 (14.3%)		37 (30.6%)	36 (13.3%)		
0	5 (1.4%)	6 (5.4%)		4 (4.4%)	2 (7.1%)					
Mean_lesion_density	−487 (155)	−432 (180)	0.005	−506 (140)	−475 (137)	0.33	−488 (141)	−400 (207)	<0.001	<0.001
CTLP	0.05 (0.08)	0.07 (0.12)	0.050	0.09 (0.15)	0.04 (0.05)	0.28	0.06 (0.11)	0.09 (0.10)	<0.001	<0.001

^1^Pearson’s Chi-squared test; Wilcoxon rank sum test; Fisher’s exact test; ^2^Pearson’s Chi-squared test; Kruskal–Wallis rank sum test. 0, adult; 1, child.

### 3.2 CT imaging signs

Statistical analysis was conducted on the CT image features of the training set, validation set, and test set. Segmental and Wedge-shaped consolidation showed significant differences between the adult group and the child group, with Segmental and Wedge-shaped consolidation in the child group, with statistical significance (*p* < 0.05; Figure 2), consolidation mixed GGO and air bronchogram signs were significantly different in children, with statistical significance (*p* < 0.05). In addition, there were statistically significant differences between adults and children in interlobular septal thickening, number of lobes involved, mean lesion density, and CTLP (*p* < 0.05), while there was no statistical difference in bronchial wall thickening (*p* > 0.05). For details, see Table 1 and Figure 2.

### 3.3 Models construction

**Figure 3:**
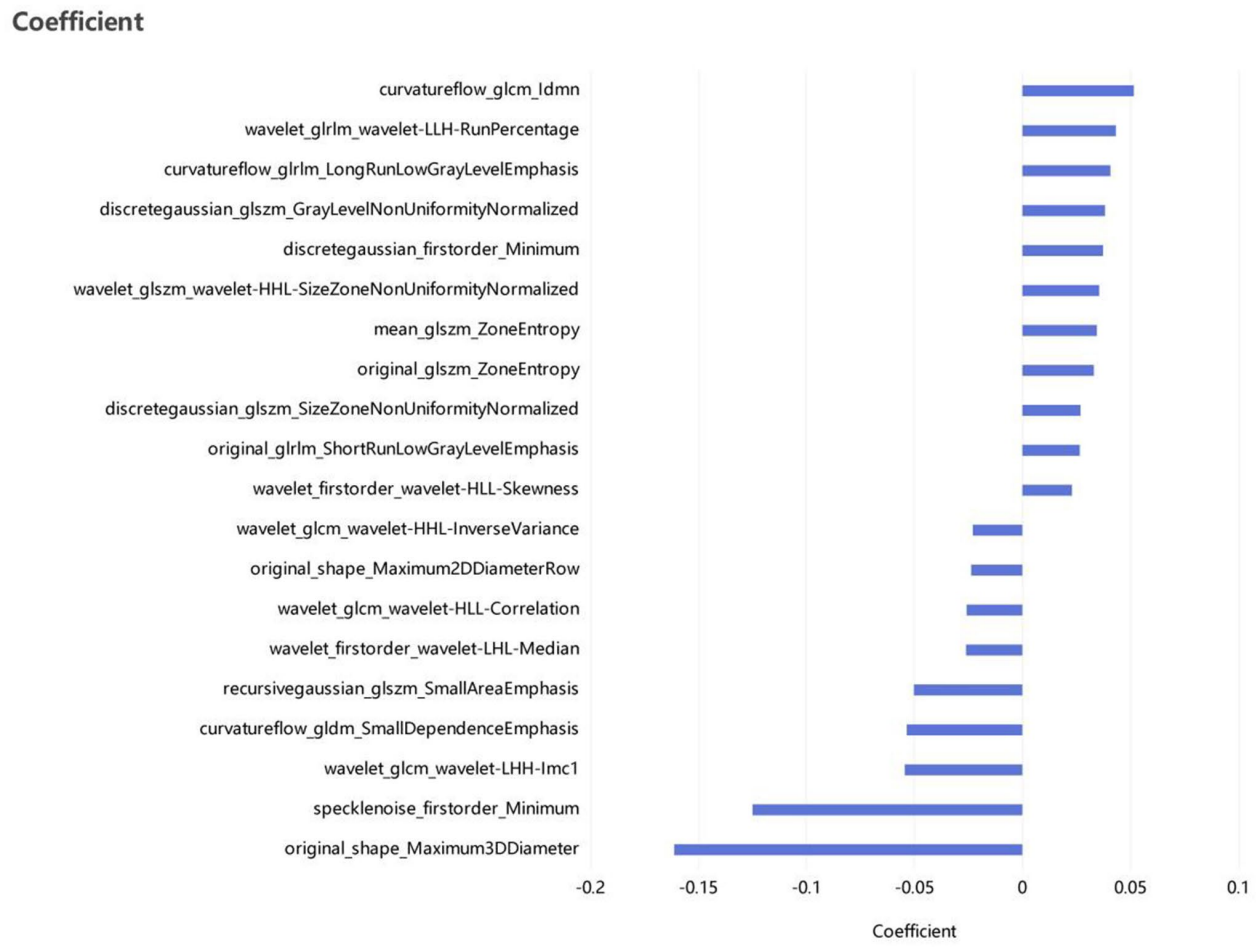
The correlation ranking of the top 20 radiomic features in the training set.

The seven most clinically relevant features extracted from the patient’s clinical characteristics are type of fever, LC, CRP, PLT, CK-MB, LDH, and D-dimer (*p* < 0.05); and 10 CT imaging signs, are consolidation pattern, consolidation mixed GGO, bronchial wall thickening, air bronchogram sign, interlobular septal thickening, number of lobes involved, pleural effusion, mediastinal enlargement of lymph nodes, mean lesion density, and CTLP, with significant differences (*p* < 0.05). Based on these features, we constructed the clinical model and the CT imaging model. For the radiomics analysis, 45 features with the highest correlation were obtained after PCC and LASSO, and Figure 3 shows the top 20 features with a correlation coefficient greater than 0.02 in the LASSO. Based on this, the radiomic model was constructed. In addition, we build the combined model using the clinical features, CT imaging signs, and the radiomics selected.

**Figure 4:**
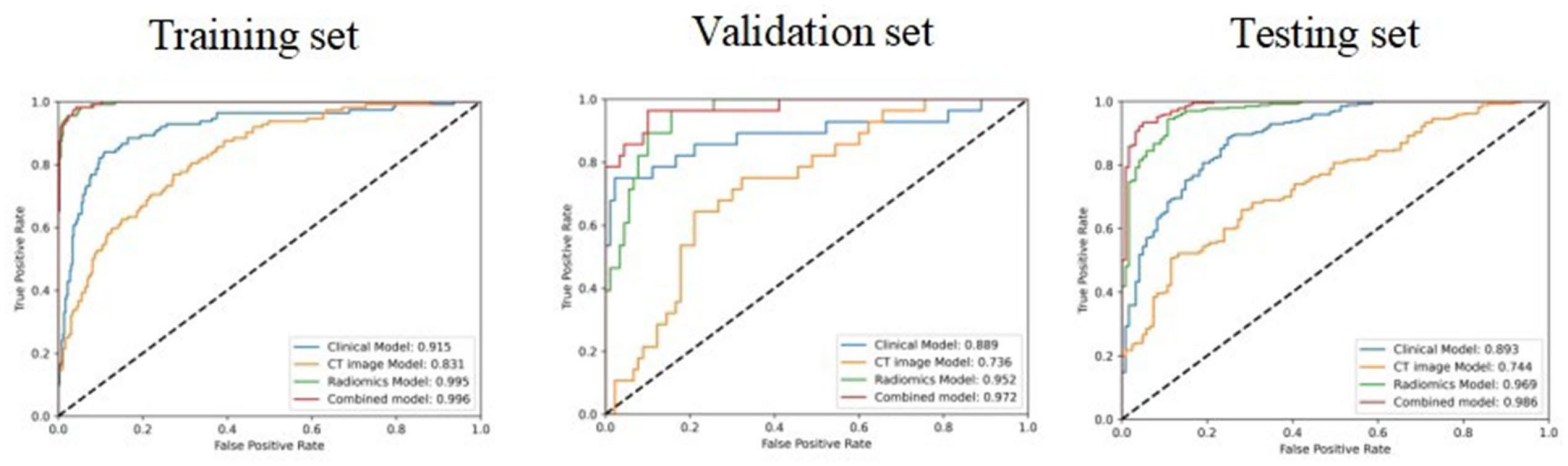
The ROC curves of the four models in the training set, validation set, and test set.

For the three models, the AUC for the testing set were 0.893(0.863,0.921), 0.744(0.698,0.783), and 0.969(0.953,0.982), the AUC for training set and validation set is shown in Table 2, and the ROC curve and prediction performance results were plotted (Table 2; Figure 4). The results showed that the combined model showed higher predictive performance in distinguishing adult and child Mycoplasma pneumonia than any single model. According to the Delong test, there was a statistical difference (*p* < 0.05) in the AUC between the CT imaging model, radiomics model, and combination model in the external test set (Table 3).

**Table 2:** Predictive ability of four models for distinguishing adult and childhood mycoplasmal pneumonia.

Model	Cohort	AUC(95% CI)	Sensitivity	Specificity	Accuracy	Precision	threshold_train
Clinical model	Training	0.915(0.886,0.941)	0.865	0.839	0.879	0.827	0.315
	Validation	0.889(0.802,0.956)	0.825	0.750	0.864	0.810	0.315
	Testing	0.893(0.863,0.921)	0.744	0.537	0.665	0.720	0.315
CT image model	Training	0.831(0.794,0.863)	0.748	0.768	0.737	0.689	0.291
	Validation	0.736(0.660,0.825)	0.714	0.750	0.695	0.659	0.291
	Testing	0.744(0.698,0.783)	0.689	0.511	0.621	0.670	0.291
Radiomics model	Training	0.995(0.992,0.998)	0.965	0.955	0.970	0.954	0.354
	Validation	0.952(0.921,0.978)	0.896	0.893	0.898	0.850	0.354
	Testing	0.969(0.953,0.982)	0.764	0.544	0.680	0.739	0.354
Combined model	Training	0.996(0.993,0.998)	0.970	0.982	0.964	0.937	0.270
	Validation	0.972(0.942,0.995)	0.890	0.857	0.907	0.864	0.270
	Testing	0.986(0.976,0.993)	0.824	0.656	0.760	0.779	0.270

**Table 3:** Comparison of AUC between the three individual models and the combined model on the test set.

Group	model_name_1	model_name_2	auc_1	auc_cov_1	auc_2	auc_cov_2	*P*-value
Test	Clinical model	CT image model	0.893	0.000307134	0.744	0.000658474	*P* > 0.05
Test	Clinical model	Radiomics model	0.893	0.000307134	0.969	7.25E-05	*P* > 0.05
Test	Clinical model	Combined model	0.893	0.000307134	0.986	2.79E-05	*P* > 0.05
Test	CT image model	Radiomics Model	0.744	0.000658474	0.969	7.25E-05	*P* < 0.05
Test	CT image model	Combined model	0.744	0.000658474	0.986	2.79E-05	*P* < 0.05
Test	Radiomics model	Combined model	0.969	7.25E-05	0.986	2.79E-05	*P* < 0.05

### 3.4 Correlation analysis between CT imaging signs and radiomics features

**Figure 5:**
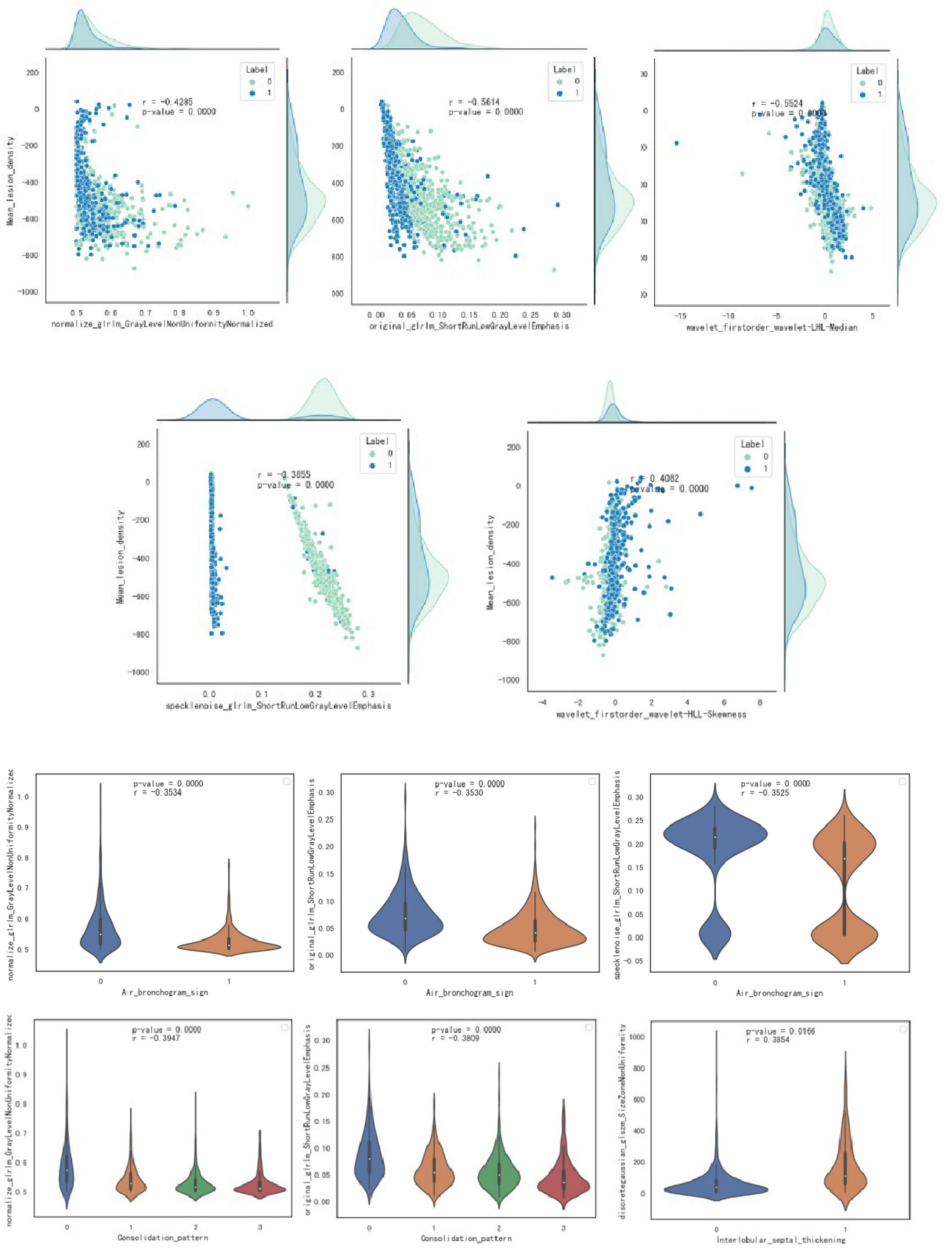
Visualization through scatter plot and box plot analysis of the correlation between quantitative and qualitative imaging features and radiomics features.

**Figure 6:**
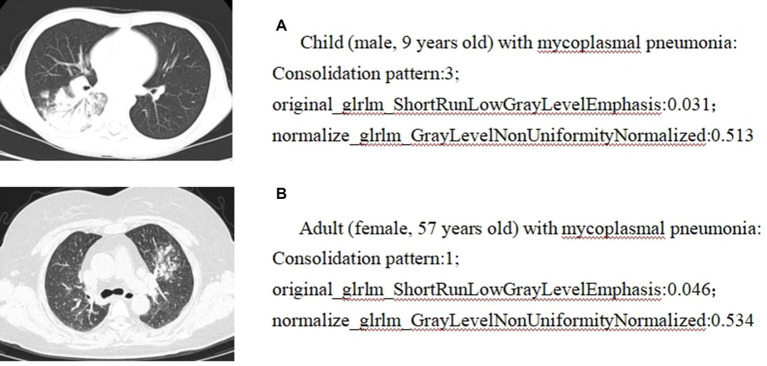
Correlation analysis between radiomics and CT imaging signs: case presentation. **(A)** Child (male, 9 years old) with mycoplasmal pneumonia: Consolidation pattern:3; original_glrlm_ShortRunLowGrayLevelEmphasis:0.031; normalize_glrlm_GrayLevelNonUniformityNormalized:0.513. **(B)** Adult (female, 57 years old) with mycoplasmal pneumonia: Consolidation pattern:1; original_glrlm_ShortRunLowGrayLevelEmphasis:0.046; normalize_glrlm_GrayLevelNonUniformityNormalized:0.534.

Pearson correlation analysis evaluated the correlation between CT features and radiomics features; the correlation map is shown in Figure 5, and the case presentation is shown in Figure 6. Those with a correlation coefficient r greater than 0.35 were included in the charts (Table 4). For the quantitative and qualitative CT images, we visualized the data distribution using box plots and correlation plots, respectively. Mean_lesion_density, Consolidation_pattern, Air_bronchogram_sign, and Interlobular_septal_thickening demonstrated a high correlation with texture features.

**Table 4:** Correlation analysis results between CT imaging signs and radiomics features.

CT feature	Radiomics feature	r	*P*
Mean_lesion_density	original_glrlm_ShortRunLowGrayLevelEmphasis	−0.561384238	1.51E-82
Mean_lesion_density	wavelet-LHL firstorder Median	−0.552387518	1.88E-79
Mean_lesion_density	normalize_glrlm_GrayLevelNonUniformityNormalized	−0.428460907	4.51E-45
Mean_lesion_density	specklenoise_glrlm_ShortRunLowGrayLevelEmphasis	−0.385539074	4.06E-36
Mean_lesion_density	wavelet-HLL firstorder Skewness	0.408192167	1.11E-40
Consolidation_pattern	original_glrlm_ShortRunLowGrayLevelEmphasis	−0.380854025	3.22E-35
Consolidation_pattern	normalize_glrlm_GrayLevelNonUniformityNormalized	−0.394684625	6.44E-38
Air_bronchogram_sign	original_glrlm_ShortRunLowGrayLevelEmphasis	−0.353032225	3.61E-30
Air_bronchogram_sign	normalize_glrlm_GrayLevelNonUniformityNormalized	−0.353370265	3.16E-30
Air_bronchogram_sign	specklenoise_glrlm_ShortRunLowGrayLevelEmphasis	−0.352479244	4.50E-30
Interlobular_septal_thickening	discretegaussian_glszm_SizeZoneNonUniformity	0.385360317	4.39E-36

## 4 Discussion

In this study, we established clinical models, CT imaging models, radiomics models, and combined models and confirmed their effectiveness in differentiating adult and children mycoplasmal pneumonia. For the individual model, the radiomics model achieved the highest AUC. In addition, the radiomics features were well correlated with CT imaging signs, which could quantitatively represent different CT imaging signs to a certain extent.

Through the analysis of the CT imaging signs of the two groups of patients, it was found that there was no or patchy consolidation in the adult group and segmental or wedge-shaped consolidation in the child group, indicating that the condition of adult Mycoplasma pneumonia was mild and slow, and children had the characteristics of rapid progress, serious disease, and high incidence of complications, which was consistent with previous studies (4). The reason for this analysis is that mycoplasma, as the smallest microorganism between bacteria and viruses, can induce cellular and humoral immune responses after infection. Due to the immature and incomplete development of the lungs in children, the number of pulmonary alveoli is relatively small compared to adults, and the immune system is relatively incomplete. The elastic fibers of the bronchial tube are not strong. After mycoplasma infection, the disease progresses faster, the function of defending inflammation is weaker, and the inflammatory manifestations are more obvious than those in adults. If it invades the bronchioles and interstitial lung tissue near the lung field, it will cause congestion, edema, infiltration, and exudation of inflammatory cells, and the exudate will stimulate the pleura, causing pleural reactive effusion, leading to pleural effusion (4). Based on the different imaging manifestations and progression of adult and children mycoplasmal pneumonia, once mycoplasmal pneumonia is diagnosed, especially in children, active treatment should be taken to prevent complications or the possibility of progression to severe disease. In addition, after feature selection, a CT imaging model was established, and a ROC curve was drawn. The internal training set AUC value was 0.831 (0.794, 0.863), the validation set AUC value was 0.736 (0.660, 0.825), and the external test set AUC value was 0.744 (0.698, 0.783). It has good discriminative power, indicating that typical CT imaging signs are important in distinguishing between adult and pediatric mycoplasmal pneumonia. At the same time, Dongdong Wang et al. (19) used radiomics to analyze the diagnostic value of distinguishing between mycoplasmal pneumonia (MPP) and *streptococcus pneumoniae* pneumonia (SPP) in children under 5 years old and divided them into a testing set and a validation set at a ratio of 7:3. In the validation cohort, the consolidation + surrounding halo sign was used to distinguish between MP and SPP, resulting in an AUC value of 0.822 and sensitivity and specificity of 0.81 and 0.81, respectively. Through the decision curve, RF was found to be superior to other classifiers.

Radiomics is an artificial intelligence technology that extracts features such as shape, intensity, texture, and wavelet from images based on images and converts them into high-dimensional quantifiable quantitative feature data to further reflect the biological information of lesions. It can provide relevant information for disease diagnosis, prognosis evaluation, and efficacy prediction (20–22). To date, few studies have used radiomics to solve the problem of pneumonia identification. Mei et al. (23) used artificial intelligence algorithms to combine chest CT findings with clinical symptoms, exposure history, and laboratory tests to diagnose COVID-19. Wang et al. (24) combined deep learning-radiomics models to distinguish COVID-19 from non-COVID-19 viral pneumonia. Honglin Li (10) confirmed that radiomics-clinical nomograms have good discriminative power for mycoplasmal pneumonia and bacterial pneumonia. These studies demonstrate the feasibility of using radiomics to identify lung inflammation. On this basis, we distinguish between adult and children mycoplasmal pneumonia. Logistic regression is a multiple regression analysis method that studies the relationship between a binary or multi-class response variable and multiple influencing factors (25). This study used the LASSO logistic regression model to screen and model 1,904 imaging features and calculated the Radscore for each patient, which can more intuitively reflect the imaging differences between adults and children with Mycoplasma pneumonia. The internal training set AUC value of the radiomics feature model in this group is 0.995 (0.992, 0.998), the validation set AUC value is 0.952 (0.921, 0.978), and the external test set AUC value is 0.969 (0.953, 0.982), indicating good differential diagnostic performance. To explore the relationship between radiomics features, CT imaging signs, and clinical features, a combined model nomogram was established based on radiomics, combining clinical and CT imaging signs. The internal training set had an AUC value of 0.996 (0.993, 0.998), the validation set had an AUC value of 0.972 (0.942, 0.995), and the external test set had an AUC value of 0.986 (0.976, 0.993), which is higher than that of the single model. Consistent with the study by Honglin Li et al. (10), a combined nomogram combining radiological and clinical features was established and validated for distinguishing Mycoplasma pneumonia and bacterial pneumonia with similar CT manifestations. In the radiomics model, the AUC of the training set was 0.877 and the AUC of the test set was 0.810. In the radiomics-clinical model, the AUC of the training set is 0.905 and the AUC of the test set is 0.847. Decision curve analysis shows that both models can improve the clinical benefits of patients, and the radiomics-clinical combination model achieves higher clinical benefits than the radiomics model.

The features of radiomics, including shape, grayscale, and texture, help to build radiomics models (26). This study establishes the correlation between radiomics features and CT imaging signs, and the study reveals that “mean lesion density” is negatively correlated with “original glrlm ShortRunLowGrayLevelEmphasis,” “wavelet-LHL firstorder Median,” “normalize glrlm GrayLevelNonUniformityNormalized,” and “specklenoise glrlm ShortRunLowGrayLevelEmphasis”; and is positively correlated with “wavelet-HLL firstorder Skewness”; “consolidation pattern” is negatively correlated with “original glrlm ShortRunLowGrayLevelEmphasis” and “normalize glrlm GrayLevelNonUniformityNormalized”; “air bronchogram sign” is negatively correlated with “original glrlm ShortRunLowGrayLevelEmphasis,” “normalize glrlm GrayLevelNonUniformityNormalized,” and “specklenoise glrlm ShortRunLowGrayLevelEmphasis”; “Interlobular_septal_thickening” is negatively correlated with “discretegaussian glszm SizeZoneNonUniformity”; and the correlation coefficients were all greater than 0.35. Most of these radiomics features are texture features and grayscale statistics features, indicating that texture features and grayscale statistics features are largely quantitative representations of CT image features. Moreover, based on the close correlation between radiomics features and traditional CT image features, the advantage of radiomics lies in its ability to transform images into a large amount of high-throughput imaging information that can be mined. Through selection and comparison of the information, optimal features are selected, resulting in more objective and accurate results (27). Radiomics is non-invasive, quantitative, easily accessible, and reproducible. When combined with CT imaging signs and clinical features, it can provide more comprehensive information about the biological characteristics and microenvironment changes of diseases and has broad prospects in disease diagnosis and prognosis evaluation. This study achieved good results in external validation, indicating that multiple centers and different scanners are beneficial for universality.

There are certain limitations in this study: (1) There are common shortcomings in retrospective studies, such as selection bias; (2) Due to the vague outline of pneumonia lesions, it is difficult to accurately delineate the ROI, and even some smaller lesions are easily missed; (3) Without classifying patients into mild and severe groups before extracting features, further research is needed to investigate the impact of different disease severities.

In summary, this study proposes that radiomics features, CT imaging signs, and clinical features facilitate the identification of differences between adults and children with mycoplasmal pneumonia. For the individual model, the radiomics model achieved the highest AUC. The radiomics features are well-correlated with CT imaging signs, which can provide a quantitative representation of different CT imaging signs using radiomics to a certain extent.

## Data availability statement

The raw data supporting the conclusions of this article will be made available by the authors, without undue reservation.

## Ethics statement

The studies involving humans were approved by the Ethics Committee of Affiliated Hospital of Hebei University Affiliated Hospital of Hebei University. The studies were conducted in accordance with the local legislation and institutional requirements. The ethics committee/institutional review board waived the requirement of written informed consent for participation from the participants or the participants' legal guardians/next of kin because since this study is a retrospective study, written informed consent is waivered.

## Author contributions

HM: Conceptualization, Data curation, Writing – original draft, Writing – review & editing. T-DW: Data curation, Writing – original draft. L-YZ: Data curation, Writing – original draft. J-WH: Writing – original draft. L-yS: Writing – review & editing. WY: Writing – original draft. L-LZ: Writing – original draft. J-JC: Data curation, Writing – review & editing. J-NW: Writing – review & editing. X-PY: Conceptualization, Resources, Supervision, Writing – review & editing.
